# Differentiation between glioma recurrence and treatment effects using amide proton transfer imaging: A mini-Bayesian bivariate meta-analysis

**DOI:** 10.3389/fonc.2022.852076

**Published:** 2022-08-01

**Authors:** Kai Chen, Xi-Wen Jiang, Li-jing Deng, Hua-Long She

**Affiliations:** ^1^ Department of Medical Imaging, Shenzhen Samii Medical Center, Shenzhen, China; ^2^ Department of Medical Imaging, Affiliated Hospital of Xiangnan University (Clinical College), Chenzhou, China; ^3^ Department of Neonatology, Shenzhen Third People’s Hospital, Second Hospital Affiliated to Southern University of Science and Technology, Shenzhen, China

**Keywords:** amide proton transfer (APT) imaging, glioma, tumor recurrence, treatment effect, pseudoprogression, radiation necrosis (RN)

## Abstract

**Background:**

Amide proton transfer (APT) imaging as an emerging MRI approach has been used for distinguishing tumor recurrence (TR) and treatment effects (TEs) in glioma patients, but the initial results from recent studies are different.

**Aim:**

The aim of this study is to systematically review and quantify the diagnostic performance of APT in assessing treatment response in patients with post-treatment gliomas.

**Methods:**

A systematic search in PubMed, EMBASE, and the Web of Science was performed to retrieve related original studies. For the single and added value of APT imaging in distinguishing TR from TEs, we calculated pooled sensitivity and specificity by using Bayesian bivariate meta-analyses.

**Results:**

Six studies were included, five of which reported on single APT imaging parameters and four of which reported on multiparametric MRI combined with APT imaging parameters. For single APT imaging parameters, the pooled sensitivity and specificity were 0.85 (95% CI: 0.75–0.92) and 0.88 (95% CI: 0.74–0.97). For multiparametric MRI including APT, the pooled sensitivity and specificity were 0.92 (95% CI: 0.85–0.97) and 0.83 (95% CI: 0.55–0.97), respectively. In addition, in the three studies reported on both single and added value of APT imaging parameters, the combined imaging parameters further improved diagnostic performance, yielding pooled sensitivity and specificity of 0.91 (95% CI: 0.80–0.97) and 0.92 (95% CI: 0.79–0.98), respectively, but the pooled sensitivity was 0.81 (95% CI: 0.65-0.93) and specificity was 0.82 (95% CI: 0.61–0.94) for single APT imaging parameters.

**Conclusion:**

APT imaging showed high diagnostic performance in assessing treatment response in patients with post-treatment gliomas, and the addition of APT imaging to other advanced MRI techniques can improve the diagnostic accuracy for distinguishing TR from TE.

## Introduction

Glioma is the most common primary brain tumor with a poor prognosis. Radiotherapy and chemotherapy are important post-treatments for glioma patients. As the treatments often produce new lesions that may mimic tumor recurrence (TR) on imaging, differentiating TR from treatment effects (TEs) remains a major clinical challenge that often leads to delay termination of ineffective therapies or premature termination of effective therapies. Hence, patients with suspected TR are frequently confirmed by stereotactic biopsy or surgery.

Tumor pseudoprogression and radiation necrosis are the major treatment-induced changes. Pseudoprogression is mainly a radiological definition, as a new or enlarging area of contrast agent enhancement, without argument of true tumor progression, which will decrease or stabilize without additional therapy (1). Pseudoprogression cases occur considerably in the first 12 weeks after the end of treatment (2), while 30% of pseudoprogression cases may occur after more than 3 months. Radiation necrosis generally occurs 3–12 months after radiotherapy, which is characterized histopathologically by fibrinoid necrosis of blood vessel walls, with adjacent perivascular parenchymal coagulative necrosis (3).

In recent years, several functional and molecular magnetic resonance (MR) imaging techniques have been applied to identify a more accurate imaging marker for tumor tissues, such as diffusion-weighted imaging (DWI), arterial spin labeling (ASL) imaging, dynamic susceptibility contrast-enhanced (DSC) imaging, MR spectroscopy (MRS), and amide proton transfer (APT) weighted imaging. Apparent diffusion coefficient (ADC) was quantified from DWI due to increased cellularity and extracellular space tortuosity. Relative cerebral blood flow (rCBF) quantified from ASL is a useful index for assessing tumor-induced neovascularization. Similar to the mechanism of rCBF, relative cerebral blood volume (rCBV) quantified from DSC seems to be a reliable technique to better identify glioma recurrence (4, 5). MRS is a molecular imaging technique that can invasively obtain information about cellular metabolism. A previous meta-analysis (6) reported that of the novel MR imaging techniques for assessing treatment response in high-grade gliomas, MRS showed the highest diagnostic accuracy; however, APT imaging was not included. APT imaging is a newly emerging molecular MR imaging technique that enables indirect measurement of endogenous mobile proteins and peptides in tissue, by detecting the magnetization transfer ratio (MTR) asymmetry at the offsets of ±3.5 ppm with respect to the water signal (7). Recent studies (8–10) have demonstrated the capability of APT imaging in assessment of post-treatment gliomas, which is a superior imaging technique to MRS (8), predominantly based on the fact that active tumors have higher protein/peptide content compared to areas of treatment-related effects due to tumor vascular endothelial damage, cytotoxicity and mutagenicity of alkylating agents, and reduced cell density (11). Positron emission tomography (PET) with the amino acid tracers is one of the most popular known to reflect protein metabolism in gliomas and has high accuracy in the diagnosis of patients with recurrent glioma (9). However, repeated radiation exposure from PET is not desirable for patients requiring long-term follow-up. Therefore, APT imaging has substantial benefit in that it uses an off-resonance radiofrequency (RF) pulse to detect endogenous mobile proteins and peptides within recurrent gliomas without ionizing radiation. Another clinical advantage of APT imaging is that it is non-invasive with no need for exogenous contrast and is a reproducible technique, which can be a potential alternative to DSC perfusion, especially in patients where contrast agent is contraindicated (12).

However, the diagnostic performance of APT imaging in assessing the response of glioma after treatment has not been systematically evaluated. Therefore, the purpose of this meta-analysis study was to evaluate the single and added value of APT imaging in differentiating TR from TE in patients with post-treatment gliomas.

## Materials and methods

This meta-analysis followed the recommendations of the Preferred Reporting Items for Systematic Reviews and Meta-Analyses to diagnostic test accuracy checklist (13). Two reviewers (Kai Chen and Xi-wen Jiang) performed the article search, study selection, data extraction, and quality assessment independently. Disagreements were resolved by consensus-based discussion with a third reviewer (Hua-Long She, with 13 years of neuroimaging experience).

### Literature search

The search process follows the guide of PICOS criteria (13). A systematic search in PubMed, Web of Science, and Embase databases was performed to find original studies relevant to the research question. We used the search query combined synonyms for glioma, APT, and TEs (see **Supplementary Table S1**). The search was performed on 2 December 2021, without a start date limit, and was restricted to studies published in English.

### Inclusion and exclusion criteria

The original research articles included were required to meet the following criteria: (1) the patients who were confirmed as gliomas on pathology examination; (2) those who had received total or subtotal tumor resection followed by either chemoradiotherapy (CCRT) or radiation therapy (RT); (3) suspected recurrent glioma on follow-up MRI; (4) used APT imaging to assess the enlarged lesion; (5) pathological or serial clinico-radiological follow-up results were used as the reference standard; (6) the true positive (TP), false positive (FP), false negative (FN), and true negative (TN) values could be extracted from articles or obtained from the authors.

Exclusion criteria were as follows: (1) studies among pediatric patients (<18 years); (2) review articles, guidelines, consensus statements, letters, editorials, and conference abstracts; (3) MRI ≤1.5 T; (4) a partially overlapping patient population; and (5) insufficient data for reconstruction of 2 × 2 tables. In the case of an overlapping study population, the study of the largest and most recent study population was included.

### Data extraction and quality assessment

Extracted data contained general characteristics (including authors, publication year, study period, total number of patients, the rate of glioma recurrence, and patient age), study characteristics (including study design, tumor histology, and time interval between post-treatment and APT imaging), MRI characteristics [including the evaluation parameters and the cutoff values, MR manufacturer, magnet field strength, MRI sequences used for APT, region of interest (ROI) selection, and combined techniques for multiparametric MRI], and key data (TP, FP, FN, and TN). When provided data were insufficient to 2 × 2 contingency tables, we contacted the corresponding author to request the original data. If the diagnostic performances of several APT parameters were separately evaluated, the results with the highest diagnostic performance were selected.

Quality Assessment of Diagnostic Accuracy Studies 2 (QUADAS-2) was used to assess the methodological quality of the included studies in Review Manager 5.3 software (Cochrane). QUADAS-2 defined quality as the risk of bias and applicability of a study in the following domains: patient selection, index tests, reference standard, flow, and timing, mainly including the level to which estimates of diagnostic accuracy avoided risk of bias, and the degree to which studies are applicable to the research question in the review (14).

### Statistical analysis

First of all, exploration of threshold effect was performed using Meta-Disc 1.4 (Ramony Cajal Hospital, Madrid, Spain) software, and then the heterogeneity of sensitivity and specificity between studies was assessed using a combination of Cochran Q and the *I* ^2^ factor with *p* < 0.05 considered significant. *I* ^2^ > 50% indicates substantial inter-study heterogeneity. As the number of studies involved was small, we used the package “meta4diag” in R version 3.6.0 (R Core Team, 2019) to perform the Bayesian approach meta-analysis. The pooled sensitivity, specificity, and their corresponding 95% confidence intervals (95% CI) were calculated to assess the diagnostic accuracy of single APT imaging parameters and its added value for multiparametric MRI, respectively. If there were overlapping data between studies, data from the largest and most appropriate study were selected for inclusion in quantitative analysis. Summarized data were represented using summary receiver operating characteristic (SROC) plots.

## Results

### Literature search

A total of 185 records were initially identified through systematic literature search. After the removal of 37 duplicates, and screening the publication type, 13 conference abstracts, 6 case reports, and 28 reviews and meta-analyses were excluded. There were 23 nonhuman studies, 69 unrelated studies, and 1 study with insufficient information to construct 2 × 2 tables. Full-text reviews were performed, and two studies that had a shared study population with the other study were excluded.

**Figure 1:**
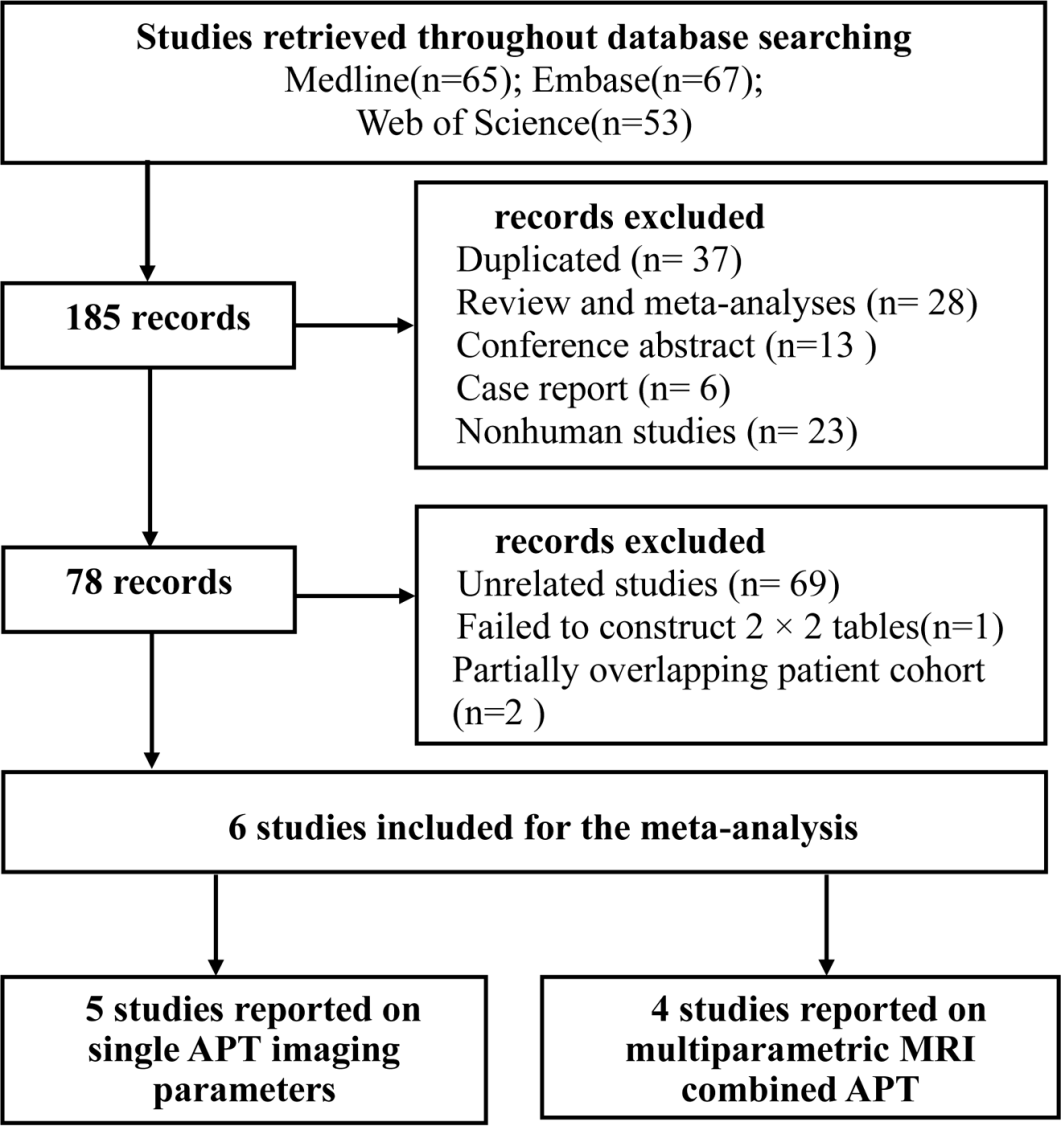
Flowchart depicting study selection.

Finally, six studies (11, 15–19) were included in the meta-analysis. Of them, five studies (11, 15, 16, 18, 19) used single APT imaging parameters and four studies (16–19) used multiparametric MRI combined with APT imaging parameters to differentiate between glioma recurrence and TEs. The detailed literature selection process is summarized in **Figure 1**.

### Characteristics of the included studies

The patient and study characteristics are described in **Table 1**. The size of the study ranged from 21 to 74 subjects, with the percentage of subjects with TR ranged from 53.33% to 85.71%. One study (16) only included patients with glioblastoma, while two studies (11, 19) included patients with high-grade gliomas (WHO III and IV). The studies using single APT imaging parameters for quantitative synthesis included a total of 184 subjects and using multiparametric MRI combined with APT imaging parameters included a total of 205 subjects. The study design was perspective in three studies (11, 17, 19) and not explicit in one study (15). Only one study (15) enrolled patients restricted to those with suspected tumor progression within the first 3 months after chemotherapy. In five studies that reported on the single value of APT (%) between TR and TE group, one study (16) used the 90% histogram intensity for the APT (APT90) as the diagnostic parameter, and the others used the mean value for the APT (APT mean).

**Table 1:** Characteristics of the included studies.

Author(year)	Study period	Design	Subjects	TR, *n* (%)	Age (years)	Tumor grade	Time interval	Diagnostic parameter	APT value (TR vs TE)	ROI selection	Cutoff value of APT	Multi-parametric MRI
Jiang S S et al. (2019) (11)	April 2010–October 2015	Pros	21	18 (85.71%)	54.6 ± 17	III–IV	353 days (43–1,311)	APTmean	2.71% ± 0.91% vs. 1.24% ± 0.29%	2–5 ROIs	1.79%	NA
Liu J et al. (2020) (19)	Unknown	Pros	30	16 (53.33%)	47.6 ± 11.4 (TR) 40.5 ± 15.0 (TE)	III–IV	20.9 ± 17.8 (weeks TR) 27.4 ± 31.9 (weeks TE)	APTmean	1.56 ± 1.14% vs. –0.44 ± 1.34%	2–5 ROIs	NA	APT + rCBF
Ma B et al. (2016) (15)	Unknown	unknown	32	20 (62.5%)	56.5 (22-78)	I–IV	3 months (1–12 months)	APTmean	2.75 ± 0.42% vs. 1.56 ± 0.42%	3–5 ROIs	2.42%	NA
Paprottka K J et al. (2021) (17)	December 2017–April 2020	Pros	74	57 (77.03%)	54.91 ± 12.2	I–IV	102 days	NA	NA	New or enlarged lesion	1.79%	APT+ rCBV
Park K J et al. (2016) (16)	August 2013–March 2015	Retro	65	37 (56.92%)	54.3(24-77)	IV	39.1 ± 11.5 (weeks TR) 57.3 ± 14.1 (weeks TE)	APT90	3.87 ± 1.72% vs. 1.38 ± 1.14%	Whole contrast-enhancing lesion	2.88%	APT + nCBV
Park Y W et al. (2021) (18)	July 2017–September 2019	Retro	36	25 (69.44%)	52.3 ± 13.7 (TR) 57.1 ± 14.8 (TE)	II–IV	77.7 ± 141.3 (weeks TR) 31.7 ± 30.1(weeks TE)	APTmean	3.18% vs. 1.77%	One ovoid ROI	2.11%	ADC + FA + nCBV + APT

Pros, prospective; Retro, retrospective; TR, tumor recurrence; TE, treatment effects; APTmean, the mean value for the APT; APT90, 90% histogram intensity for the APT; T, Tesla; NA, not available; ROI, region of interest; ADC, apparent diffusion coefficient; rCBV, relative cerebral blood volume; nCBV, normalized cerebral blood volume; rCBF, relative cerebral blood flow; FA, fractional anisotropy.

Time interval: interval between completion of post-treatment and APT imaging.

In the four studies that reported on multiparametric MRI, three studies (16–18) used DSC imaging, including two studies (16, 18) that used normalized rCBV (nCBV) as a parameter. The rCBV was normalized by dividing the rCBV value in the region of interest by the rCBV value of contralateral side (5). One study (18) also combined DWI and DTI, using ADC and fractional anisotropy (FA) as parameters. In addition, one study (19) only combined rCBF quantified from ASL.

APT imaging uses a series of frequency-selective RF pulses tuned at 3.5 ppm upfield of the water resonance labeling the amide protons. APT signal intensity is reported as a percentage change in the bulk water signal intensity, which depends on the pulse sequence features and parameters used. According to the recent consensus (20), we summarized the MR hardware and APT imaging techniques, as shown in **Table 2**. The Philips Achieva 3.0-T MR scanner was the most used device in five studies. In the pulse sequence of APT imaging, the pulse train was used in four studies as RF saturation. 3D pulse sequence readout was used for image acquisition in five studies and 3D gradient and spin-echo (3D-GRASE) was the most used sequence. Only one study (19) used 2D single-shot spin-echo planar imaging (SE-EPI) readout to acquire images, which had the lowest values of APT (1.56 ± 1.14% TR vs. –0.44 ± 1.34% TE).

**Table 2:** APT imaging technique of the included studies using MTR asymmetry (at 3.5 ppm) on MRI systems.

Author (year)	Hardware	Pulse sequence				
		RF saturationapproach	RF saturationparameters		Readout	Acquisitionprotocol
Jiang S S et al. (2019) (11)	Philips Achieva 3.0 T	Pulse train	t_p_ = 200 ms, t_d_ = 10 ms, *n* = 4, DCsat = 95%, Tsat = 830 ms, B1 = 2 μT	3D-GRASE		Z-spectrum
Liu J et al. (2020) (19)	GE, Discovery MR750 3.0 T	Pulse train	t_p_ = 400 ms, t_d_ = 0 ms, *n* = 3, DCsat = 100%, Tsat = 1.2 s, B1 = 1.5 μT	Single-slice, SE-EPI		Z-spectrum
Ma B et al. (2016) (15)	Philips Achieva 3.0 T	Pulse train	tp = 200 ms, t_d_ = 10 ms, *n* = 4, DCsat = 95%, Tsat = 830 ms, B1 = 2 μT	3D-GRASE		Z-spectrum
Paprottka K J et al. (2021) (17)	Philips Achieva or Ingenia 3.0 T	Time-interleaved pTX	t_p_ = 50 ms, t_d_ = 0 ms, *n* = 40, DCsat = 100%, Tsat = 2 s, B1 = 2 μT	3D-FSE		6-offset
Park K J et al. (2016) (16)	Philips Achieva 3.0 T	Time-interleaved pTX	t_p_ = 70 ms, t_d_ = 70 ms, *n* = 30, DCsat = 50%, Tsat = 4.2 s, B1 = 1 μT	3D-GRE		Z-spectrum
Park Y W et al. (2021) (18)	Philips Achieva or Ingenia 3.0 T	Pulse train	t_p_ = 200 ms, t_d_ = 0 ms, *n* = 4, DCsat = 100%, Tsat = 800 ms, B1 = 2 μT		3D-GRASE	6-offset

pTX, parallel transmit; t_p_, individual pulse element duration in a pulse train; t_d_, interpulse delay; n, number of pulse element-delay repetitions; DCsat, saturation duty cycle (= t_p_/[t_p_ + t_d_]); Tsat, total RF saturation time; B1, RF saturation field strength.

Z-spectrum, normalized water saturation signal (S_sat_/S_0_) as a function of frequency offset relative to the water resonance, where S_sat_ and S_0_ are water signal intensities with and without RF saturation, respectively; 6-offset, APT MRI saturation at the saturation frequency offsets ( ± 3.0, ± 3.5, ± 4.0 ppm) from water and without saturation, for example.

SE, spin-echo acquisition; FSE, fast spin echo; EPI, echo planar imaging; GRE, gradient echo; GRASE, gradient and spin-echo acquisition.

### Quality assessment

**Figure 2:**
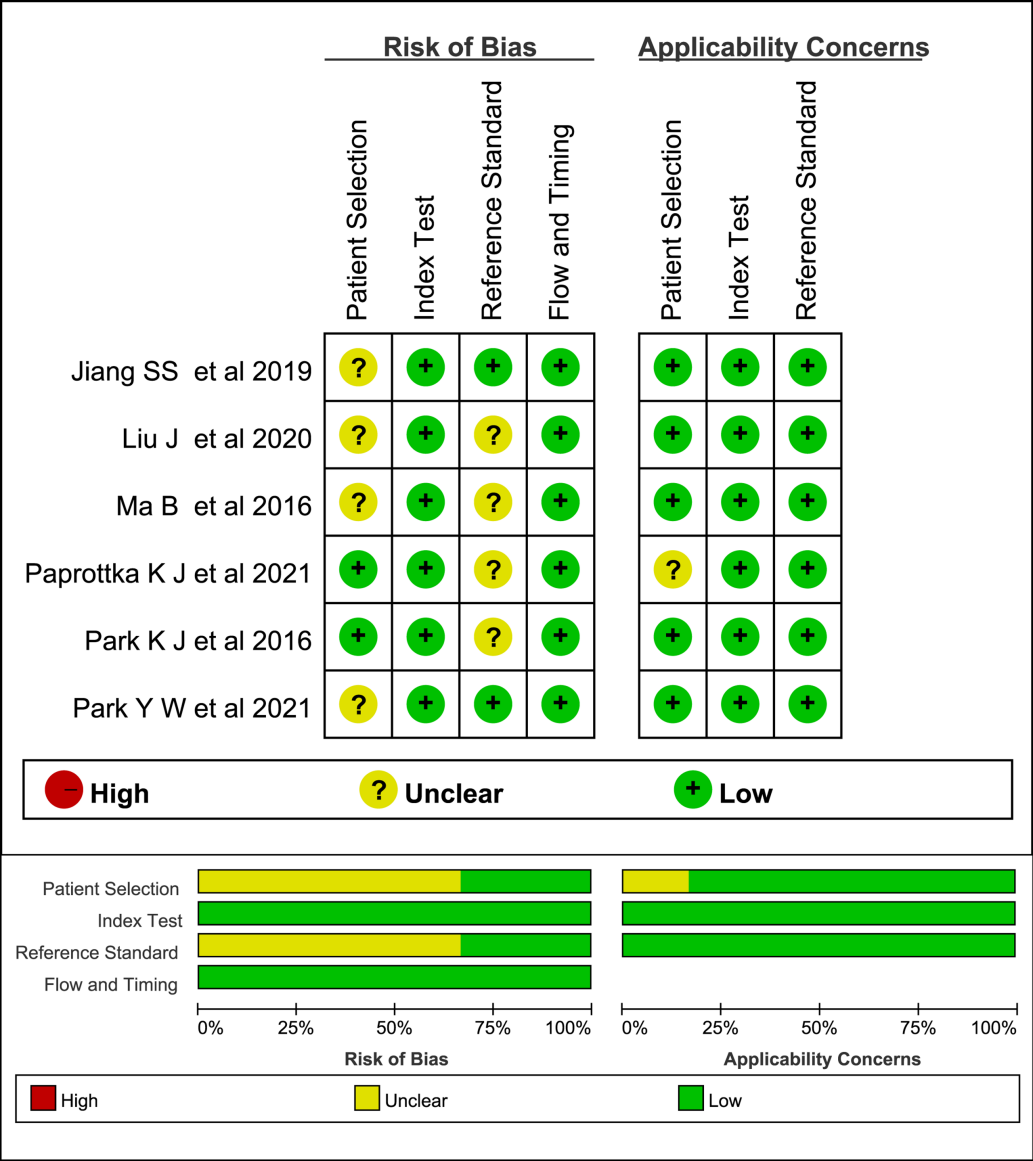
Stacked bar charts of Quality Assessment of Diagnostic Accuracy Studies 2 scores of methodologic study quality.

The QUADAS-2 scores of each study are presented in **Figure 2**. Overall, all included studies had a low to unclear risk of bias and minimal concerns regarding applicability. In the first domain regarding patient selection, four studies (11, 15, 18, 19) had unclear risk of bias due to concerns about not explicitly mentioning whether patient enrollment was consecutive. In the index test domain, all studies had a low risk of bias. Four studies (15–17, 19) had an unclear risk of bias for the reference standard domain, because it was unmentioned whether the results of the reference standard were interpreted without knowledge of the index test. In the flow and timing domain, all studies had a low risk of bias.

Regarding the applicability assessment, one study (17) had an unclear applicability concern in patient selection, as 4/74 cases did not receive radiotherapy. We had no concerns that the conduct and interpretation of the index test and the reference standard do not match our review questions in any of the studies.

### Data analysis

The results of the diagnostic threshold analysis demonstrated that no significant threshold effect existed.

**Figure 3:**
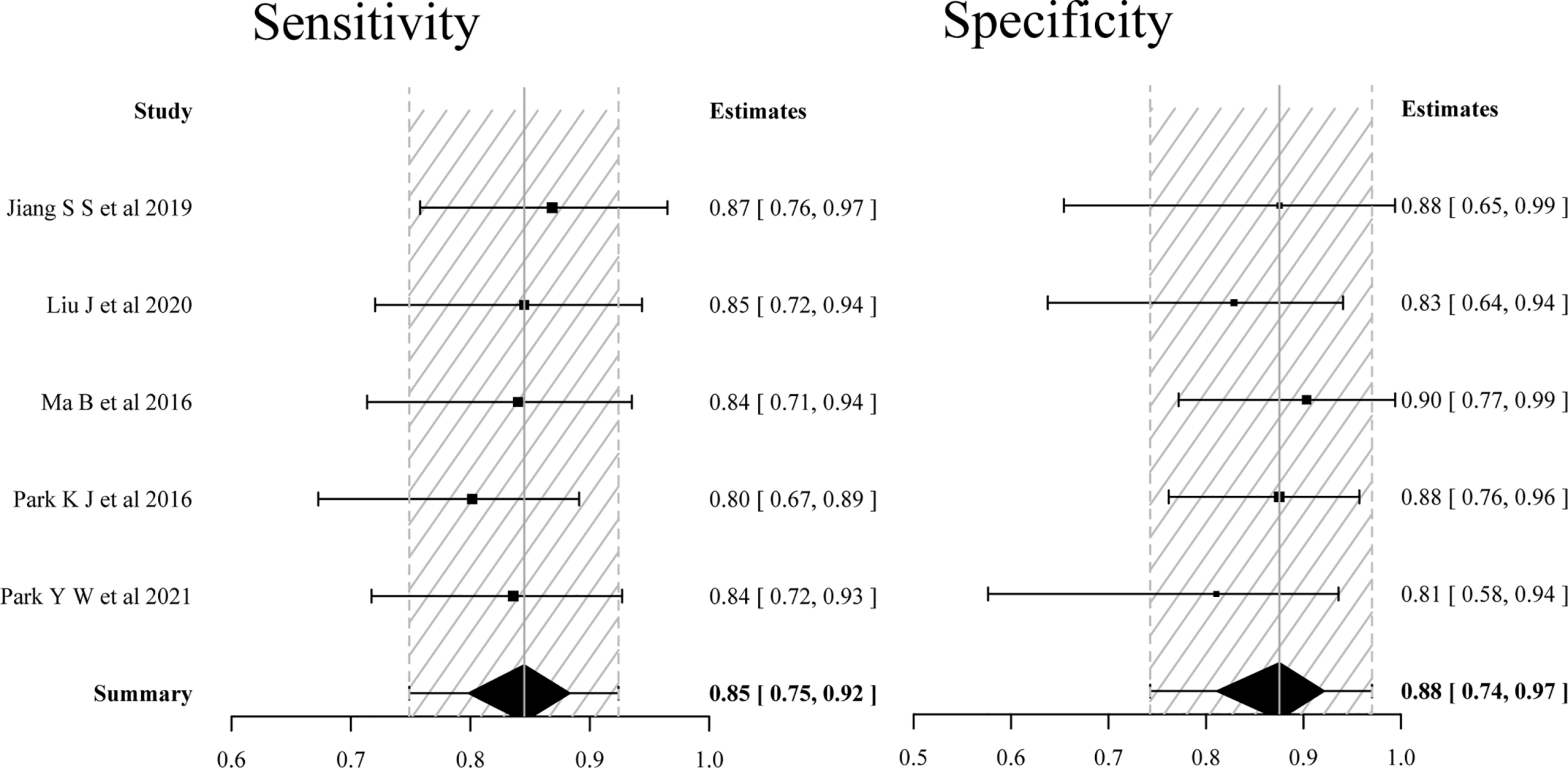
Forest plots of the sensitivity and specificity of single APT imaging parameters for differentiating tumor recurrence and treatment effects in patients with post-treatment glioma.

The five studies using the single APT imaging parameters to differentiate TR from TE showed no significant heterogeneity in sensitivity (*p* = 0.445, *I* ^2^ = 0%) and limited heterogeneity in specificity (*p* = 0.156, *I* ^2^ = 39.8%). The estimated sensitivities and specificities of the five included individual studies were 0.80–0.87 and 0.81–0.90, respectively. The pooled sensitivity and specificity were 0.85 (95% CI: 0.75–0.92) and 0.88 (95% CI: 0.74–0.97), respectively. The forest plot demonstrated mild heterogeneity between studies (**Figure 3**); in addition, the SROC curve (**Figure 5A**) showed a difference between the 95% credible region and prediction region. The estimated sensitivities were higher in studies with a smaller total sample of patients (*N* < 50), and estimated specificities were also slightly higher in the two studies (11, 15) that used 3D gradient and spin-echo (GRASE) APT imaging sequence.

**Figure 4:**
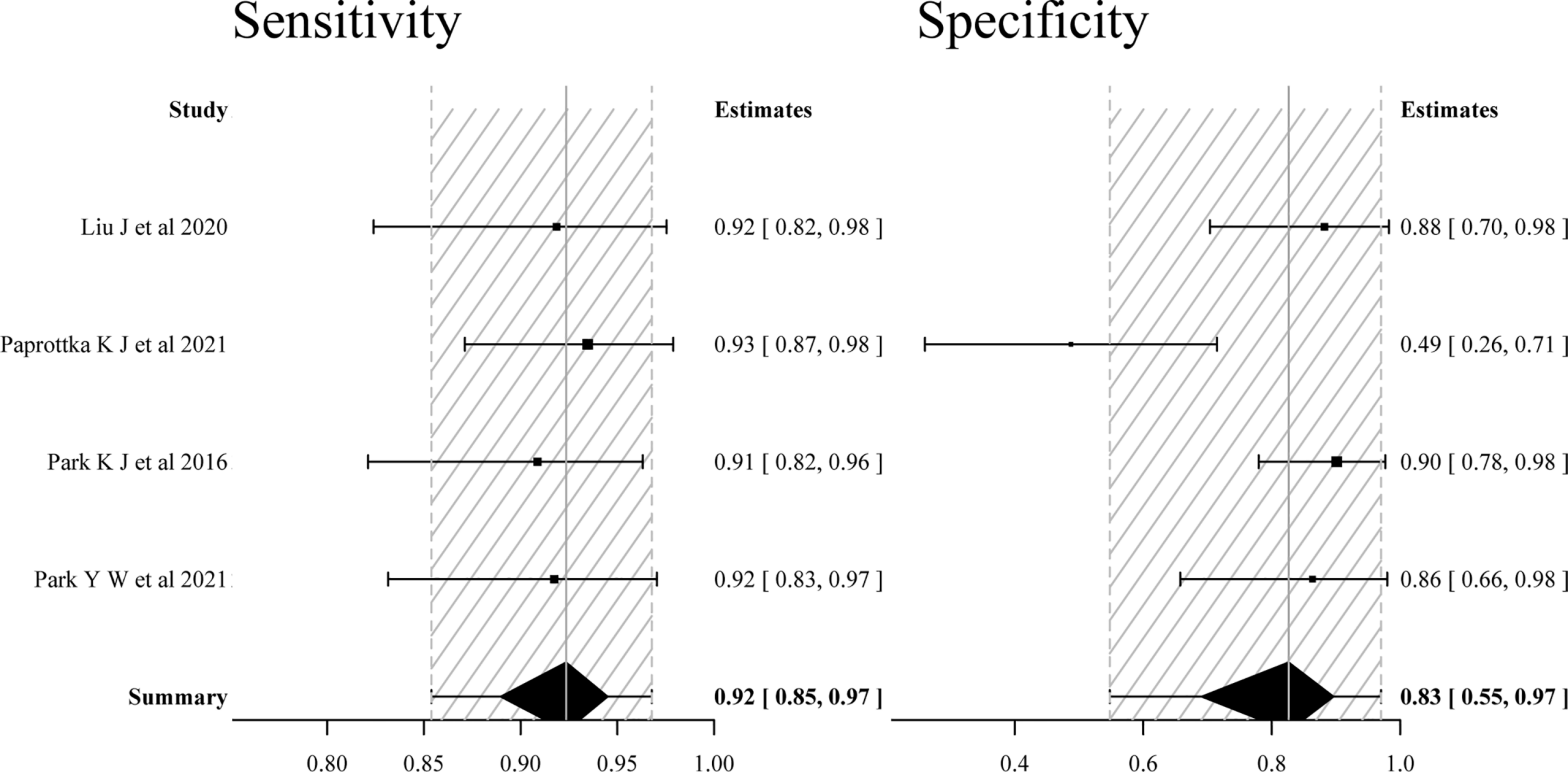
Forest plots of the sensitivity and specificity of multiparametric MRI including APT imaging parameters for differentiating tumor recurrence and treatment effects in patients with post-treatment glioma.

**Figure 5:**
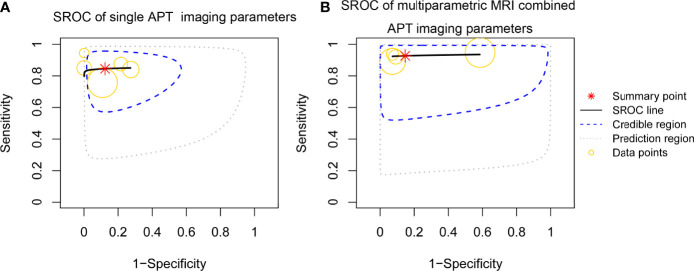
Summary receiver operating characteristic (SROC) curve of the diagnostic performance of single APT imaging parameters and multiparametric MRI including APT imaging parameters for differentiating tumor recurrence and treatment effects in patients with post-treatment glioma. **(A)** SROC curve of single APT imaging parameters (estimate of AUC was 0.863). **(B)** SROC curve of multiparametric MRI including APT imaging parameters (estimate of AUC was 0.933).

**Figure 6:**
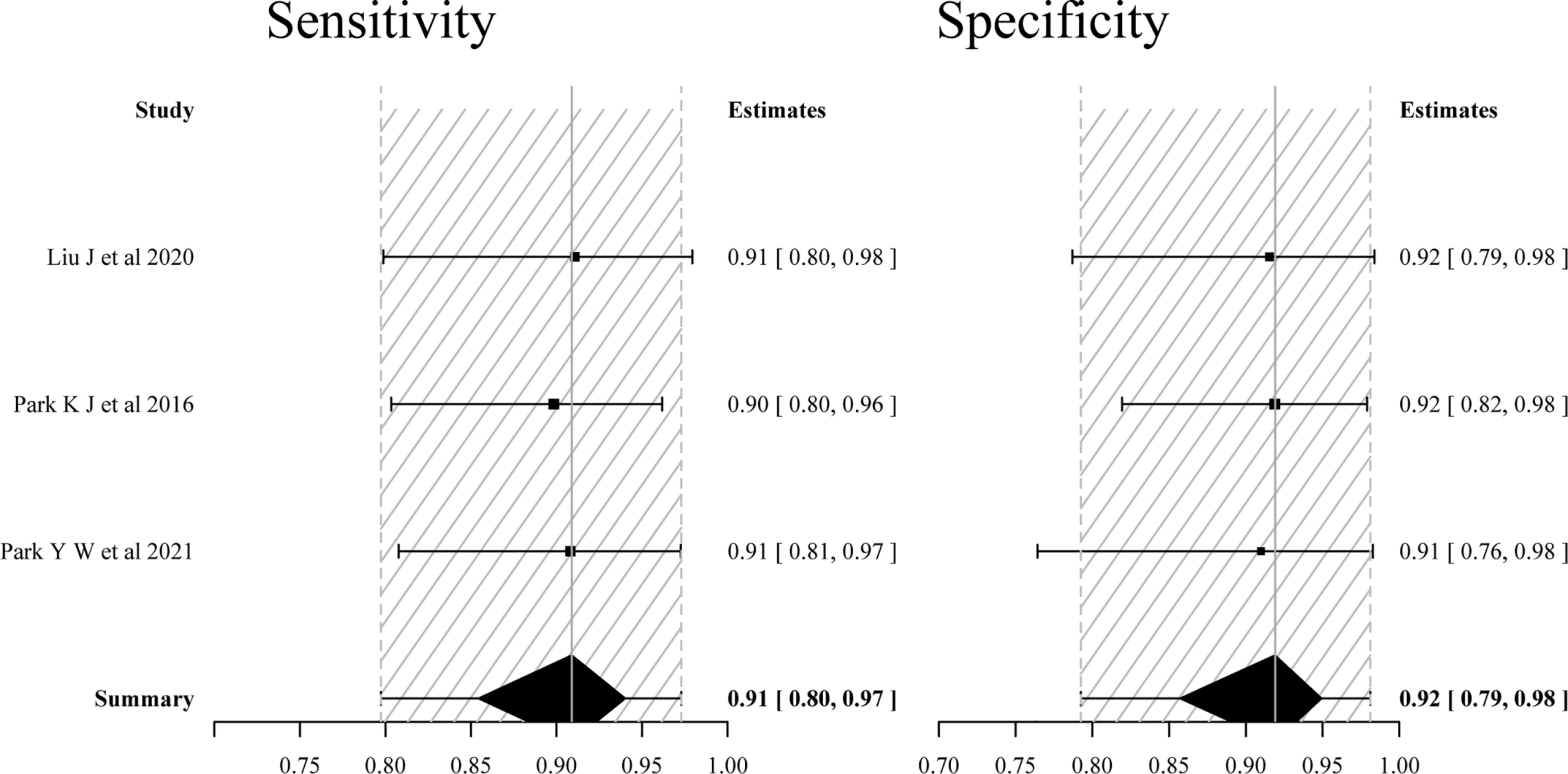
Forest plots of the sensitivity and specificity of multiparametric MRI including APT imaging parameters (only included three studies reported on both the single and added value of APT imaging parameters) for differentiating tumor recurrence and treatment effects in patients with post-treatment glioma.

The four studies (16–19) using multiparametric MRI combined with APT imaging parameters to differentiate TR from TE showed no significant heterogeneity in sensitivity (*p* = 0.796, *I* ^2^ = 0%), but substantial heterogeneity in specificity (*p* < 0.001, *I* ^2^ = 84.0%). The pooled sensitivity and specificity of the four studies were 0.92 (95% CI: 0.85–0.97) and 0.83 (95% CI: 0.55–0.97), respectively. The forest plot (**Figure 4**) and the SROC plot (**Figure 5B**) showed that the primary source of heterogeneity was the study (17) that has a slightly higher sensitivity and extremely low specificity. With the exception of that study, the remaining three studies (16, 18, 19) showed no significant heterogeneity in sensitivity (*p* = 0.846, *I* ^2^ = 0%) and specificity (*p* = 0.978, *I* ^2^ = 0%). Moreover, the three studies had both reported on single APT imaging parameters and multiparametric MRI combined with APT in differentiating TR from TE, and APT parameters added to multiparametric MRI improved diagnostic performance, yielding pooled sensitivity and specificity of 0.91 (95% CI: 0.80–0.97) and 0.92 (95% CI: 0.79–0.98), respectively (**Figure 6**), while the pooled sensitivity was 0.81 (95% CI: 0.65–0.93) and specificity was 0.82 (95% CI: 0.61–0.94) for single APT imaging parameters (**Figure S1**).

## Discussion

In this meta-analysis, we evaluated the value of APT imaging in the evaluation of post-treatment response in glioma patients. Our results indicate that APT imaging is an exciting prospect in distinguishing glioma recurrence from TE, especially when combined with other multiparametric MRI parameters.

Compared to the previous mate analysis (6), we found that the diagnostic accuracy of APT imaging was similar to the nCBV derived from DSC and higher than the ADC derived from DWI, but was lower than MRS, both of which were commonly used imaging biomarkers in multiparametric MRI for the determination of treatment response after chemoradiotherapy in patients with glioblastoma (5). However, these were indirect comparisons, and only allowing all techniques to be tested in the same population would overcome the main limitation.

Mixed response in post-treatment glioma can result from a variety of nontumorous processes, including ischemia, postsurgical changes, treatment-related inflammation, subacute radiation effects, and radiation necrosis (17). rCBV and rCBF were useful indices for assessing tumor-induced neovascularization. Nevertheless, inflammation can also lead to increased value. On DWI, recurrent tumors may show reduced ADC due to increased cellularity and extra-cellular space tortuosity, but radiation necrosis may also show diffusion restriction, presumably due to intracellular edema and viscous material in the transition zone (21). Consequently, increased rCBV and rCBF does not always mean viable tumor angiogenesis, and decreased ADC values do not always mean high cellularity. Various metabolic ratios were used in the MRS studies. However, choline (Cho)/creatine (Cr) peak-area ratio was identified as the best predictor in identifying recurrent glioma after the post-treatment (6). Cho peak is one of the most important indicators in evaluating brain tumor proliferation. Cr peak maintains certain stability in the development of many diseases; thus, it is often used as a reference. In practice, MRS is more technically challenging and the voxel sizes are relatively large. Ideally, any technique that can reliably detect glioma proliferation within a larger area of TEs should cover the entire radiation volume.

APT imaging provides different regional information and increases the diagnostic value for multiparametric MRI, while the best cutoff values for the advance MRI techniques precisely distinguishing post TR from TE were arbitrary because of the heterogeneity in the biological activity of glioma and the use of different MRI systems. Of the four studies using multiparametric MRI combined with APT, one study (17) used the predefined thresholds from the literature by using the following cutoff values: APT > 1.79 (11) and rCBV > 5.6 (22), resulting in a slight increase in sensitivity, but a significant decrease in specificity. After excluding the study, the heterogeneities across the three studies in terms of pooled sensitivity and specificity were significantly reduced (**Figure 4**), whereas the diagnostic performance was not significantly superior to the results reported in a previous meta-analysis (5), which used multiparametric MRI but not combined with APT, with a pooled sensitivity and specificity of 0.84 (95% CI: 0.74–0.91) and 0.95 (95% CI: 0.83–0.99), respectively. However, the fact that meta-analysis only evaluated the value of multiparametric MRI for the determination of early treatment response, the studies containing mixed TE cases, such as pseudoprogression occurring after more than 3 months and radiation necrosis, were excluded. A potential source of heterogeneity among the studies using single APT imaging parameters to differentiate TR from TE might be the use of different sequences. One study used 2D SE-EPI sequence, which has low signal intensity and small differences between TR and TE groups, while relatively high estimated sensitivity and specificity were obtained in the studies using a 3D GRASE sequence. The 3D GRASE APT imaging technique has been confirmed to have reliable image quality and reasonable scan time (23).

This mate analysis used the Bayesian approach. Bayesian inference adds a small amount of informative priors that can stabilize the analysis without overwhelming data (24), as opposed to the frequentist methods that express the initial uncertainty with a prior distribution. Bayesian bivariate meta-analyses have advantages in estimating the heterogeneity among studies and pooled effect, especially when the number of studies included is small (25).

The limitations of this study include a relatively small number of studies. In addition, the mean intervals between the end of post-treatment and APT imaging were varied among studies, leading to the inclusion of several stages of treatment-related changes. However, a previous meta-analysis found no difference between early follow-up studies and studies that were conducted more than 3 months after CCRT (6). Additionally, we did not evaluate the publication bias, and another study raised a similar concern about the studies using APT in differentiating TR from TE, which consistently report positive results (26). Lastly, the repeatability of APT signal was excellent in supratentorial locations, while it was poor in infratentorial locations due to severe B0 inhomogeneity and susceptibility, which affects MTR asymmetry (27), and the locations of the glioma were not mentioned in the included studies. Nevertheless, caution is required when applying our results to daily clinical practice.

## Conclusion

This mini-Bayesian bivariate meta-analysis disclosed that APT imaging has high diagnostic performance in evaluating treatment response in patients with post-treatment gliomas, and the addition of APT imaging to other advanced MRI techniques can improve the diagnostic accuracy for distinguishing TR from TE. However, based on the current evidence from a small number of studies, further evaluation is required.

## Data availability statement

The original contributions presented in the study are included in the article/**Supplementary Material**. Further inquiries can be directed to the corresponding author.

## Author contributions

H-LS contributed to the study design, KC and X-WJ contributed to literature search, collection, and assembly of data. Data analysis was performed by KC and L-JD. KC wrote the first draft of the manuscript. H-LS and L-JD revised and edited the manuscript. All authors read and approved the final version.

## Funding

This work was supported by the Science and Technology Planning Project of Chenzhou City, Hunan Province [zdyf 201973 and zdyf 20201811].

## Conflict of interest

The authors declare that the research was conducted in the absence of any commercial or financial relationships that could be construed as a potential conflict of interest.

## Publisher’s note

All claims expressed in this article are solely those of the authors and do not necessarily represent those of their affiliated organizations, or those of the publisher, the editors and the reviewers. Any product that may be evaluated in this article, or claim that may be made by its manufacturer, is not guaranteed or endorsed by the publisher.

## Supplementary material

Supplementary Figure 1 Forest plots of the sensitivity and specificity of single APT imaging parameters (only included three studies reported on both the single and added value of APT imaging parameters) for differentiating tumor recurrence and treatment effects in patients with post-treatment glioma

## References

[1] Garcia GCTE Dhermain F . Pseudoprogression in gliomas: the use of advanced MRI for treatment decisions. Curr Treat Options Neurol (2020) 22:23. doi: 10.1007/s11940-020-00630-8

[2] Wen PY Macdonald DR Reardon DA Cloughesy TF Sorensen AG Galanis E . Updated response assessment criteria for high-grade gliomas: response assessment in neuro-oncology working group. J Clin Oncol (2010) 28(11):1963–72. doi: 10.1200/jco.2009.26.3541 20231676

[3] Zikou A Sioka C Alexiou GA Fotopoulos A Voulgaris S Argyropoulou MI . Radiation necrosis, pseudoprogression, pseudoresponse, and tumor recurrence: Imaging challenges for the evaluation of treated gliomas. Contrast Media Mol Imaging (2018) 2018:6828396. doi: 10.1155/2018/6828396 30627060 PMC6305027

[4] Wang S Martinez-Lage M Sakai Y Chawla S Kim SG Alonso-Basanta M . Differentiating tumor progression from pseudoprogression in patients with glioblastomas using diffusion tensor imaging and dynamic susceptibility contrast MRI. AJNR Am J Neuroradiol (2016) 37(1):28–36. doi: 10.3174/ajnr.A4474 26450533 PMC7960225

[5] Suh CH Kim HS Jung SC Choi CG Kim SJ . Multiparametric MRI as a potential surrogate endpoint for decision-making in early treatment response following concurrent chemoradiotherapy in patients with newly diagnosed glioblastoma: a systematic review and meta-analysis. Eur Radiol (2018) 28:2628–38. doi: 10.1007/s00330-017-5262-5 29374321

[6] van Dijken BRJ van Laar PJ Holtman GA van der Hoorn A . Diagnostic accuracy of magnetic resonance imaging techniques for treatment response evaluation in patients with high-grade glioma, a systematic review and meta-analysis. Eur Radiol (2017) 27:4129–44. doi: 10.1007/s00330-017-4789-9 PMC5579204 28332014

[7] Sotirios B Demetriou E Topriceanu CC Zakrzewska Z . The role of APT imaging in gliomas grading: A systematic review and meta-analysis. Eur J Radiolo (2020) 133:109353. doi: 10.1016/j.ejrad.2020.109353 33120241

[8] Park JE Kim HS Park KJ Kim SJ Kim JH Smith SA . Pre- and posttreatment glioma: Comparison of amide proton transfer imaging with MR spectroscopy for biomarkers of tumor proliferation. Radiology (2016) 278(2):514–23. doi: 10.1148/radiol.2015142979 26491847

[9] Park JE Lee JY Kim HS Oh JY Jung SC Kim SJ . Amide proton transfer imaging seems to provide higher diagnostic performance in post-treatment high-grade gliomas than methionine positron emission tomography. Eur Radiol (2018) 28(8):3285–95. doi: 10.1007/s00330-018-5341-2 29488086

[10] Meissner J-E Korzowski A Regnery S Goerke S Breitling J Floca RO . Early response assessment of glioma patients to definitive chemoradiotherapy using chemical exchange saturation transfer imaging at 7 T. J Magnet Reson Imaging (2019) 50(4):1268–77. doi: 10.1002/jmri.26702 30864193

[11] Jiang SS Eberhart CG Lim M Heo HY Zhang Y Blair L . Identifying recurrent malignant glioma after treatment using amide proton transfer-weighted MR imaging: A validation study with image-guided stereotactic biopsy. Clin Cancer Res (2019) 25(2):552–61. doi: 10.1158/1078-0432.Ccr-18-1233 PMC6335169 30366937

[12] Friismose AI Markovic L Nguyen N Gerke O Schulz MK Mussmann BR . Amide proton transfer-weighted MRI in the clinical setting–correlation with dynamic susceptibility contrast perfusion in the post-treatment imaging of adult glioma patients at 3T. Radiography (2022) 28(1):95–101. doi: 10.1016/j.radi.2021.08.006 34509365

[13] Moher D Shamseer L Clarke M Ghersi D Liberati A Petticrew M . Preferred reporting items for systematic review and meta-analysis protocols (PRISMA-p) 2015 statement. Syst Rev (2015) 4(1):1. doi: 10.1186/2046-4053-4-1 25554246 PMC4320440

[14] Whiting PF Rutjes AW Westwood ME Mallett S Deeks JJ Reitsma JB . QUADAS-2: a revised tool for the quality assessment of diagnostic accuracy studies. Ann Intern Med (2011) 155(8):529–36. doi: 10.7326/0003-4819-155-8-201110180-00009 22007046

[15] Ma B Blakeley JO Hong X Zhang H Jiang S Blair L . Applying amide proton transfer-weighted MRI to distinguish pseudoprogression from true progression in malignant gliomas. J Magnet Reson Imaging (2016) 44(2):456–62. doi: 10.1002/jmri.25159 PMC4946988 26788865

[16] Park KJ Kim HS Park JE Shim WH Kim SJ Smith SA . Added value of amide proton transfer imaging to conventional and perfusion MR imaging for evaluating the treatment response of newly diagnosed glioblastoma. Eur Radiol (2016) 26(12):4390–403. doi: 10.1007/s00330-016-4261-2 26883333

[17] Paprottka KJ Kleiner S Preibisch C Kofler F Schmidt-Graf F Delbridge C . Fully automated analysis combining f-18 -FET-PET and multiparametric MRI including DSC perfusion and APTw imaging: a promising tool for objective evaluation of glioma progression. Eur J Nucl Med Mol Imaging (2021) 48(13):4445–55. doi: 10.1007/s00259-021-05427-8 PMC8566389 34173008

[18] Park YW Ahn SS Kim EH Kang SG Chang JH Kim SH . Differentiation of recurrent diffuse glioma from treatment-induced change using amide proton transfer imaging: incremental value to diffusion and perfusion parameters. Neuroradiology (2021) 63(3):363–72. doi: 10.1007/s00234-020-02542-5 32879995

[19] Liu J Li C Chen Y Lv X Lv Y Zhou J . Diagnostic performance of multiparametric MRI in the evaluation of treatment response in glioma patients at 3T. J Magn Reson Imaging (2020) 51(4):1154–61. doi: 10.1002/jmri.26900 31430008

[20] Zhou J Zaiss M Knutsson L Sun PZ Ahn SS Aime S . Review and consensus recommendations on clinical APT-weighted imaging approaches at 3T: Application to brain tumors. Magn Reson Med (2022) 88(2):546–74. doi: 10.1002/mrm.29241 PMC9321891 35452155

[21] Verma N Cowperthwaite MC Burnett MG Markey MK . Differentiating tumor recurrence from treatment necrosis: a review of neuro-oncologic imaging strategies. Neuro Oncol (2013) 15(5):515–34. doi: 10.1093/neuonc/nos307 PMC3635510 23325863

[22] Göttler J Lukas M Kluge A Kaczmarz S Gempt J Ringel F . Intra-lesional spatial correlation of static and dynamic FET-PET parameters with MRI-based cerebral blood volume in patients with untreated glioma. Eur J Nucl Med Mol Imaging (2017) 44(3):392–7. doi: 10.1007/s00259-016-3585-0 27913827

[23] Zhou J Zhu H Lim M Blair L Quinones-Hinojosa A Messina SA . Three-dimensional amide proton transfer MR imaging of gliomas: Initial experience and comparison with gadolinium enhancement. J Magnet Reson Imaging (2013) 38(5):1119–28. doi: 10.1002/jmri.24067 PMC3664658 23440878

[24] Guo J Riebler A and Rue H . Bayesian Bivariate meta-analysis of diagnostic test studies with interpretable priors. Stat Med (2017) 36(19):3039–58. doi: 10.1002/sim.7313 28474394

[25] Martin E Geitenbeek RTJ Coert JH Hanff DF Graven LH Grünhagen DJ . A Bayesian approach for diagnostic accuracy of malignant peripheral nerve sheath tumors: a systematic review and meta-analysis. Neuro Oncol (2021) 23(4):557–71. doi: 10.1093/neuonc/noaa280 PMC8041346 33326583

[26] Okuchi S Hammam A Golay X Kim M and Thust S . Endogenous chemical exchange saturation transfer MRI for the diagnosis and therapy response assessment of brain tumors: A systematic review. Radiol Imaging Cancer (2020) 2(1):e190036. doi: 10.1148/rycan.2020190036 33778693 PMC7983695

[27] Akbari H Kazerooni AF Ware JB Mamourian E Anderson H Guiry S . Quantification of tumor microenvironment acidity in glioblastoma using principal component analysis of dynamic susceptibility contrast enhanced MR imaging. Sci Rep (2021) 11(1):15011. doi: 10.1038/s41598-021-94560-3 34294864 PMC8298590

