# Supplementary material for: Differentiation between glioma recurrence and treatment effects using amide proton transfer imaging: A mini-Bayesian bivariate meta-analysis
Source: Front Oncol. 2022 Aug 1;12:852076. doi: 10.3389/fonc.2022.852076 (PMC9376615; doi:10.3389/fonc.2022.852076)
Supplement: Supplementary Figure 1 — Forest plots of the sensitivity and specificity of single APT imaging parameters (only included three studies reported on both the single and added value of APT imaging parameters) for differentiating tumor recurrence and treatment effects in patients with post-treatment glioma [file DataSheet_1.docx]

Supplementary Table 1: Search syntaxes

Supplementary Table 2: Diagnostic accuracy of characteristics not in meta-analyses

**Supplementary Table 1 Search syntaxes**

| **PubMed**  **2-Dec-2021** | (("glioma"[MeSH Terms] OR "glioma"[All Fields] OR "gliomas"[All Fields] OR "glioma s"[All Fields] OR ("astrocytoma"[MeSH Terms] OR "astrocytoma"[All Fields] OR "astrocytomas"[All Fields]) OR "glioblastom"[All Fields] OR "astrocytom"[All Fields] OR "oligodendrogliom"[All Fields]) AND ("apt"[Supplementary Concept] OR "apt"[All Fields] OR "apt"[All Fields] OR (("amidate"[All Fields] OR "amidated"[All Fields] OR "amidates"[All Fields] OR "amidating"[All Fields] OR "amidation"[All Fields] OR "amidations"[All Fields] OR "amidative"[All Fields] OR "amides"[MeSH Terms] OR "amides"[All Fields] OR "amide"[All Fields] OR "amidic"[All Fields] OR "etomidate"[MeSH Terms] OR "etomidate"[All Fields] OR "etomidate s"[All Fields]) AND ("proton s"[All Fields] OR "protonable"[All Fields] OR "protonate"[All Fields] OR "protonated"[All Fields] OR "protonates"[All Fields] OR "protonating"[All Fields] OR "protonation"[All Fields] OR "protonations"[All Fields] OR "protonic"[All Fields] OR "protonics"[All Fields] OR "protons"[MeSH Terms] OR "protons"[All Fields] OR "proton"[All Fields]) AND ("transfer"[All Fields] OR "transferability"[All Fields] OR "transferable"[All Fields] OR "transfered"[All Fields] OR "transfering"[All Fields] OR "transferred"[All Fields] OR "transferring"[All Fields] OR "transfers"[All Fields])) OR "CEST"[All Fields]) AND ((("therapeutics"[MeSH Terms] OR "therapeutics"[All Fields] OR "treatments"[All Fields] OR "therapy"[MeSH Subheading] OR "therapy"[All Fields] OR "treatment"[All Fields] OR "treatment s"[All Fields]) AND ("effect"[All Fields] OR "effecting"[All Fields] OR "effective"[All Fields] OR "effectively"[All Fields] OR "effectiveness"[All Fields] OR "effectivenesses"[All Fields] OR "effectives"[All Fields] OR "effectivities"[All Fields] OR "effectivity"[All Fields] OR "effects"[All Fields])) OR (("therapeutics"[MeSH Terms] OR "therapeutics"[All Fields] OR "treatments"[All Fields] OR "therapy"[MeSH Subheading] OR "therapy"[All Fields] OR "treatment"[All Fields] OR "treatment s"[All Fields]) AND ("change"[All Fields] OR "changed"[All Fields] OR "changes"[All Fields] OR "changing"[All Fields] OR "changings"[All Fields])) OR (("therapeutics"[MeSH Terms] OR "therapeutics"[All Fields] OR "treatments"[All Fields] OR "therapy"[MeSH Subheading] OR "therapy"[All Fields] OR "treatment"[All Fields] OR "treatment s"[All Fields]) AND ("change"[All Fields] OR "changed"[All Fields] OR "changes"[All Fields] OR "changing"[All Fields] OR "changings"[All Fields])) OR (("therapeutics"[MeSH Terms] OR "therapeutics"[All Fields] OR "treatments"[All Fields] OR "therapy"[MeSH Subheading] OR "therapy"[All Fields] OR "treatment"[All Fields] OR "treatment s"[All Fields]) AND ("response"[All Fields] OR "responses"[All Fields] OR "responsive"[All Fields] OR "responsiveness"[All Fields] OR "responsivenesses"[All Fields] OR "responsives"[All Fields] OR "responsivities"[All Fields] OR "responsivity"[All Fields])) OR (("therapeutical"[All Fields] OR "therapeutically"[All Fields] OR "therapeuticals"[All Fields] OR "therapeutics"[MeSH Terms] OR "therapeutics"[All Fields] OR "therapeutic"[All Fields]) AND ("response"[All Fields] OR "responses"[All Fields] OR "responsive"[All Fields] OR "responsiveness"[All Fields] OR "responsivenesses"[All Fields] OR "responsives"[All Fields] OR "responsivities"[All Fields] OR "responsivity"[All Fields])) OR ("response"[All Fields] OR "responses"[All Fields] OR "responsive"[All Fields] OR "responsiveness"[All Fields] OR "responsivenesses"[All Fields] OR "responsives"[All Fields] OR "responsivities"[All Fields] OR "responsivity"[All Fields]) OR ("post"[All Fields] AND ("therapeutics"[MeSH Terms] OR "therapeutics"[All Fields] OR "treatments"[All Fields] OR "therapy"[MeSH Subheading] OR "therapy"[All Fields] OR "treatment"[All Fields] OR "treatment s"[All Fields])) OR "pseudoprogression"[All Fields] OR "pseudorespons"[All Fields] OR (("cysts"[MeSH Terms] OR "cysts"[All Fields] OR "cyst"[All Fields] OR "neurofibroma"[MeSH Terms] OR "neurofibroma"[All Fields] OR "neurofibromas"[All Fields] OR "tumor s"[All Fields] OR "tumoral"[All Fields] OR "tumorous"[All Fields] OR "tumour"[All Fields] OR "neoplasms"[MeSH Terms] OR "neoplasms"[All Fields] OR "tumor"[All Fields] OR "tumour s"[All Fields] OR "tumoural"[All Fields] OR "tumourous"[All Fields] OR "tumours"[All Fields] OR "tumors"[All Fields]) AND ("recurrance"[All Fields] OR "recurrence"[MeSH Terms] OR "recurrence"[All Fields] OR "recurrences"[All Fields] OR "recurrencies"[All Fields] OR "recurrency"[All Fields] OR "recurrent"[All Fields] OR "recurrently"[All Fields] OR "recurrents"[All Fields])) OR (("radiate"[All Fields] OR "radiated"[All Fields] OR "radiates"[All Fields] OR "radiating"[All Fields] OR "radiation"[MeSH Terms] OR "radiation"[All Fields] OR "electromagnetic radiation"[MeSH Terms] OR ("electromagnetic"[All Fields] AND "radiation"[All Fields]) OR "electromagnetic radiation"[All Fields] OR "radiations"[All Fields] OR "radiation s"[All Fields] OR "radiator"[All Fields] OR "radiators"[All Fields]) AND ("necrose"[All Fields] OR "necrosed"[All Fields] OR "necrosi"[All Fields] OR "necrosing"[All Fields] OR "necrosis"[MeSH Terms] OR "necrosis"[All Fields] OR "necroses"[All Fields])))) AND (english[Filter]) |
| --- | --- |
| **Embase**  **2-Dec-2021** | ((glioma or astrocytoma or glioblastom or astrocytom or oligodendrogliom or oligoastrocytom) and (APT or Amide proton transfer or CEST) and (treatment effect or treatment change or treatment changes or treatment response or therapeutic response or response or post treatment or pseudoprogression or pseudorespons or tumour recurrence or radiation necrosis)).af |
| **Web of Science**  **2-Dec-2021** | TS= (glioma OR astrocytoma OR glioblastom OR astrocytom OR oligodendrogliom OR oligoastrocytom) AND TS=(APT OR amide proton transfer OR CEST OR Nuclear Overhauser Enhancement) AND TS= (treatment effect OR treatment change OR treatment response OR therapeutic response OR response OR post treatment OR pseudoprogression OR pseudorespons OR tumour recurrence OR radiation necrosis) |
|  |  |

**Supplementary Table 2 Diagnostic accuracy of characteristics not in meta-analyses**

|  |  |  |  |  |  | **Accuracies (95% CI)** | |
| --- | --- | --- | --- | --- | --- | --- | --- |
| **Author**  **(year)** | **N** | **TP** | **FP** | **FN** | **TN** | Sensitivity | Specificity |
| **Single APT imaging parameters** | | | | | | | |
| Jiang S S  et al(2019) | 21 | 17 | 0 | 1 | 3 | 0.94(0.73-1.00) | 1.00(0.29-1.00) |
| Liu J et al  (2020) | 30 | 14 | 3 | 2 | 11 | 0.88(0.62-0.98) | 0.79(0.49-0.95) |
| Ma B et al  (2016) | 32 | 17 | 0 | 3 | 12 | 0.85(0.62-0.97) | 1.00(0.74-0.98) |
| Park K J et al (2016) | 65 | 28 | 3 | 9 | 25 | 0.76(0.59-0.88) | 0.89(0.72-0.98) |
| Park Y W et al(2021) | 36 | 21 | 3 | 4 | 8 | 0.84(0.64-0.95) | 0.73(0.39-0.94) |
| **Multiparametric MRI including APT imaging parameters** | | | | | | | |
| Liu J et al  (2020) | 30 | 15 | 1 | 1 | 13 | 0.94(0.70-1.00) | 0.93(0.66-1.00） |
| Paprottka K J et al  (2021) | 74 | 54 | 10 | 3 | 7 | 0.95(0.85-0.99) | 0.41(0.18-0.67) |
| Park K J et al (2016) | 65 | 33 | 2 | 4 | 26 | 0.89(0.75-0.97) | 0.93(0.76-0.99) |
| Park Y W et al(2021) | 36 | 23 | 1 | 2 | 10 | 0.92(0.74-0.99) | 0.91(0.59-1.00) |


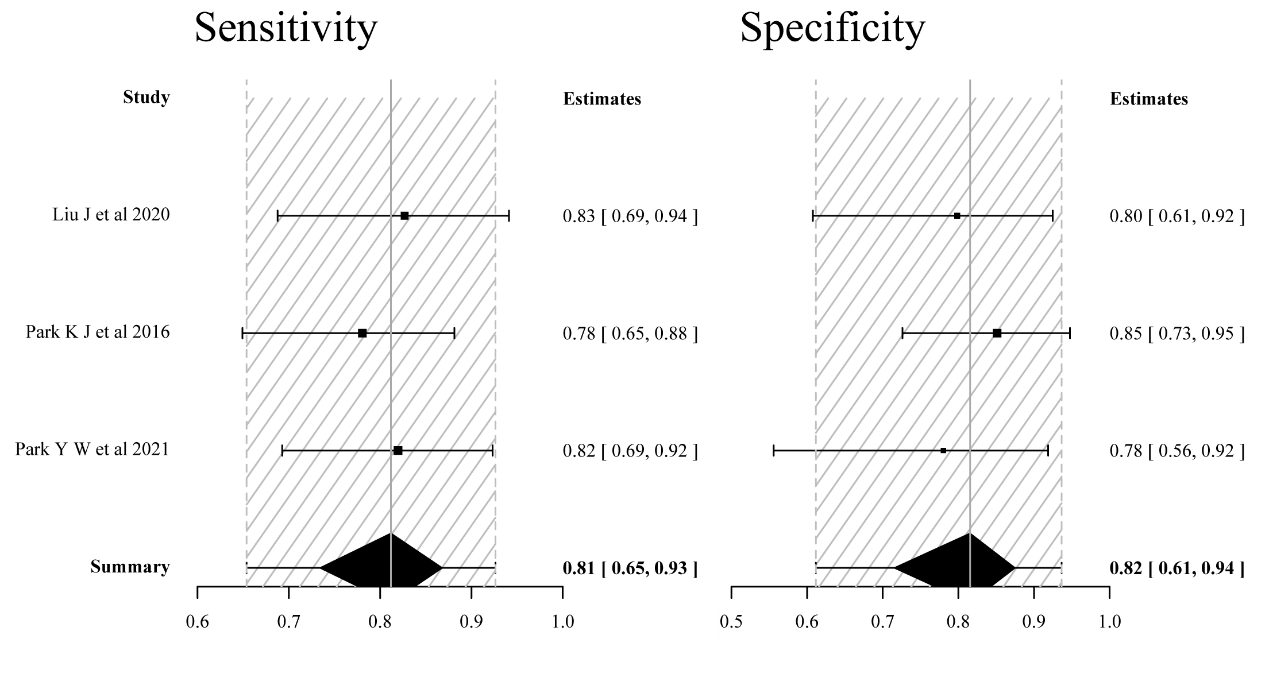


**Figure 1**

Forest plots of the sensitivity and specificity of single APT imaging parameters (only included three studies reported on both the single and added value of APT imaging parameters) for differentiating tumor recurrence and treatment effects in patients with post-treatment glioma.
